# Japanese Herbal Kampo Hochu-Ekki-To or Juzen-Taiho-To after Surgery for Hip Fracture Does Not Reduce Infectious Complications

**DOI:** 10.1155/2018/8620198

**Published:** 2018-04-29

**Authors:** Yusuke Sasabuchi, Hiroki Matsui, Alan Kawarai Lefor, Taisuke Jo, Nobuaki Michihata, Kiyohide Fushimi, Hideo Yasunaga

**Affiliations:** ^1^Data Science Center, Jichi Medical University, Tochigi, Japan; ^2^Department of Clinical Epidemiology and Health Economics, The University of Tokyo, Tokyo, Japan; ^3^Department of Surgery, Jichi Medical University, Tochigi, Japan; ^4^Department of Health Services Research, Graduate School of Medicine, The University of Tokyo, Tokyo, Japan; ^5^Department of Health Policy and Informatics, Tokyo Medical and Dental University, Graduate School of Medicine, Tokyo, Japan

## Abstract

**Background:**

Infectious complications after hip fracture surgery are common in the elderly. Although experimental studies have suggested that kampo medicine, Hochu-ekki-to and Juzen-taiho-to, can prevent infectious complications, only a few small clinical studies have been published to date.

**Primary Study Objective:**

The aim of the present study is to investigate the impact of Hochu-ekki-to or Juzen-taiho-to on postoperative infectious complications in patients undergoing surgery for hip fracture.

**Methods and Design:**

In this retrospective cohort study using a nationwide inpatient database in Japan, we performed propensity score matching to compare patients who did or did not receive kampo medicine after surgery for hip fracture.

**Settings:**

A nationwide inpatient database.

**Participants:**

Patients who did or did not receive kampo medicine after surgery for hip fracture.

**Intervention:**

Kampo medicine after surgery for hip fracture.

**Primary Outcome Measures:**

Infectious complications.

**Results:**

The proportions of postoperative infectious complications were not significantly different between the 424 propensity-matched pairs with and without kampo medicine (11 versus 8, *P* = 0.644).

**Conclusion:**

The present study suggests that Hochu-ekki-to or Juzen-taiho-to postoperatively is not associated with decreased occurrence of infectious complications in patients who underwent surgery for hip fracture.

## 1. Introduction

Hip fractures are common in the elderly. With an aging population, the incidence of hip fractures is increasing [1, 2]. The incidence of hip fractures worldwide is estimated to be 2.6 million by 2025 and to exceed 7 million by 2050 [3]. Pneumonia and surgical site infections are common complications after surgery for hip fracture [4, 5]. These complications have been reported to increase mortality after surgery for hip fracture [6, 7].

Japanese herbal kampo medicines have become popular in Japan [8]. Hochu-ekki-to and Juzen-taiho-to are two major kampo medicines, which are used to treat immunocompromised individuals [9, 10]. Experimental studies have suggested that Hochu-ekki-to and Juzen-taiho-to can prevent infectious diseases through enhancing the immune system [11–15]. Although Hochu-ekki-to and Juzen-taiho-to may be promising to prevent infectious diseases, only a few small clinical studies [16–21] have been published to date.

The aim of the present study is to investigate the impact of Hochu-ekki-to or Juzen-taiho-to on postoperative infectious complications in patients undergoing surgery for hip fracture, using a nationwide inpatient database in Japan.

## 2. Materials and Methods

The Institutional Review Board of the University of Tokyo approved this study. Informed consent was waived due to the anonymous nature of the data.

### 2.1. Data Source

Patient data were extracted from the Diagnosis Procedure Combination database, which is a nationwide administrative claims database with discharge abstracts representing approximately 50% of all admissions to acute care hospitals in Japan [22, 23]. The Diagnosis Procedure Combination database provides (i) patient demographic data, (ii) admission-precipitating diagnosis, preexisting comorbidities on admission, and complications during hospitalization coded with the International Classification of Diseases, Tenth Revision (ICD-10) codes, (iii) hospital identification number, (iv) dates of procedures performed and dates of medications administered, (v) discharge status, and (vi) dates of hospital or intensive care unit admission and discharge. Physicians in charge record data for diagnoses, comorbidities, and discharge status.

### 2.2. Patients

In the present study, patients were included if they (i) had a hip fracture (ICD-10 S720, S721, or S722) as the admission-precipitating diagnosis, (ii) underwent surgery (open reduction and internal fixation or hemiarthroplasty) for hip fracture, and (iii) were discharged between July 2010 and March 2014. Exclusion criteria included the following: (i) age < 40 years; (ii) body mass index data being unavailable, less than 10 kg/m^2^ or more than 70 kg/m^2^; (iii) number of hospital beds being not available.

### 2.3. Exposure of Interest, Outcomes, and Other Variables

The exposure of interest was whether patients received Hochu-ekki-to or Juzen-taiho-to within seven days of surgery. These patients were defined as the kampo group. Other patients were defined as the control group.

Assessed outcomes include infectious complications and in-hospital deaths. Infectious complications include surgical site infection (SSI, ICD-10, T793, or T814), hospital acquired pneumonia (J10–18), and sepsis (A40 or A41).

Other variables evaluated include age, gender, body mass index, comorbidities extracted using algorithms developed by Quan et al. [24], fracture location, type of surgery, days between admission and the surgery, treatment year, the volume of red cell transfusion, and anesthesia time. As an index for severity, use of mechanical ventilation, administration of catecholamines, administration of red cell transfusion, renal replacement therapy, and admission to a high intensity care unit such as an intensive care unit or high care unit within seven days of surgery are also extracted.

### 2.4. Statistical Analysis

Continuous variables are presented as the average with the standard deviation. Categorical variables are presented as the number with a percentage. Differences in the baseline characteristics between the kampo group and control group are compared using standardized differences. A standardized difference < 10% indicates a negligible imbalance in baseline characteristics between groups [25].

To estimate the probability that a patient would receive Hochu-ekki-to or Juzen-taiho-to medicine, a propensity score was calculated for each patient using a multivariable logistic regression for receiving kampo medicine. As independent variables, the baseline characteristics shown in Table 1 are incorporated. Patients who received kampo medicine were 1-to-1 matched with patients in the control group on the basis of nearest neighbor matching without replacement. The caliper was set at 20% of the standard deviation of the propensity score. Outcomes between the two groups were compared using Fisher's exact test. A *P* value < 0.05 is considered statistically significant. Propensity score matching is performed using “Matching” package of R version 3.1.3 (The R Foundation, Vienna, Austria). All other analyses are performed using SPSS version 22 (SPSS Inc., Chicago, Illinois, US).

## 3. Results

**Figure 1:**
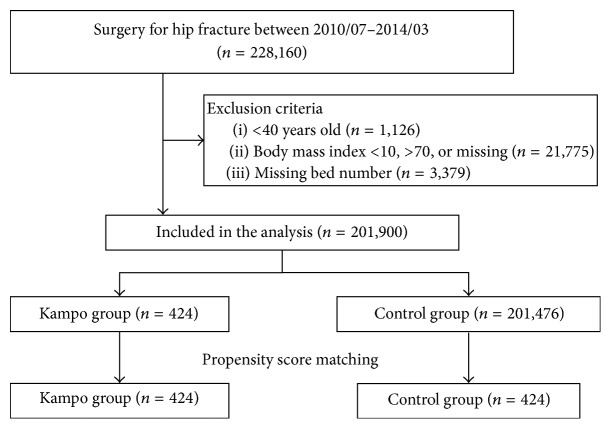
Study flow diagram.

After inclusion and exclusion criteria were applied, a total of 201,900 patients were included in the analysis (Figure 1). Of these, 424 patients received Hochu-ekki-to (259 patients) or Juzen-taiho-to (165 patients) within seven days of surgery.

Table 1 shows a comparison of baseline characteristics of patients in the kampo group and the control group before and after propensity score matching. Before propensity score matching, patients in the kampo group were more likely to have chronic pulmonary disease, peptic ulcer, and malignancy. Anesthesia time was shorter and the amount of transfusion was less in the kampo group. After propensity score matching, baseline characteristics between the two groups are well balanced.

Table 2 shows outcomes comparing the two groups. After propensity score matching, neither infectious complications nor in-hospital death was significantly different between the groups.

## 4. Discussion

In this retrospective study using a nationwide administrative database in Japan, postoperative use of Hochu-ekki-to or Juzen-taiho-to was not associated with decreased occurrence of postoperative infectious complications or in-hospital death for patients undergoing surgery for hip fracture.

Previous animal studies have suggested immunostimulating properties of Hochu-ekki-to and Juzen-taiho-to. However, only a few small clinical studies have been conducted. Hochu-ekki-to prevented immunosuppression induced by surgery [17] or inhibited rhinovirus infection in airway epithelium [18]. Clinical studies of Juzen-taiho-to have shown improved host immunity in patients with brain tumors [21] and a significantly decreased frequency of otitis media was in children [19, 20].

There are several possible explanations why the results of the present study failed to show a favorable effect of the drugs. First, the timing of administration of the kampo medicine may not be appropriate. In the present study, the kampo group received Hochu-ekki-to or Juzen-taiho-to within seven days of surgery, whereas patients received kampo medicine before surgery in a previous study [17]. Postoperative administration of kampo medicine may not sufficiently reduce postoperative infectious complications in patients undergoing surgery for hip fracture. Second, previous studies mainly included infection-prone patients. Although the patients in the present study were older (mean age more than 80 years), most of these patients may have been immunocompetent. Hochu-ekki-to or Juzen-taiho-to may be effective in immunocompromised patients. Lastly, the lack of a significant reduction in infective complications may be due to type II error. Further study, including larger numbers of patients, is warranted.

We acknowledge that the present study has several limitations. First, despite the use of propensity score matching, residual confounding may bias the results because of the retrospective nature of the study. Second, the study does not show a preventive effect for infectious complications of kampo medicine; however, the sample size may not be sufficiently large to detect a difference between the groups. Third, the database does not contain information before admission or after discharge. Patients may have had Hochu-ekki-to or Juzen-taiho-to before surgery or may have suffered from infectious complications after discharge. Fourth, patients who received Hochu-ekki-to or Juzen-taiho-to were from 238 of 1147 hospitals. The fact that patients who received these kampo medicines were from a relatively small fraction of hospitals may bias the results. Fifth, antibiotic usage was not accounted for in estimating the propensity score because it was difficult to differentiate antibiotics use for prevention from use for treatment.

In conclusion, postoperative Hochu-ekki-to or Juzen-taiho-to is not associated with a decrease in the occurrence of infectious complications after surgery for hip fracture. It is possible that receiving Hochu-ekki-to or Juzen-taiho-to only after surgery is not sufficient to prevent postoperative complications.

## Figures and Tables

**Table 1:** Baseline characteristics before and after propensity score matching.

	Prematching cohort			Propensity score matched cohort		
	Control	Kampo	SMD	Control	Kampo	SMD
	*n* = 201476	*n* = 424		*n* = 424	*n* = 424	
Age, years, mean (SD)	82.1 (9.5)	83.0 (8.0)	0.110	83.2 (8.8)	83.0 (8.0)	0.022
Gender, (Female)	158327 (78.6)	332 (78.3)	0.007	334 (78.8)	332 (78.3)	0.011
BMI, kg/m^2^, mean (SD)	20.6 (3.6)	19.7 (3.6)	0.247	19.6 (3.4)	19.7 (3.6)	0.029
Charlson comorbidity						
Myocardial infarction	2702 (1.3)	3 (0.7)	0.063	8 (1.9)	3 (0.7)	0.104
Congestive heart disease	15246 (7.6)	30 (7.1)	0.019	30 (7.1)	30 (7.1)	<0.001
Peripheral vascular disease	2766 (1.4)	10 (2.4)	0.073	9 (2.1)	10 (2.4)	0.016
Cerebrovascular disease	20739 (10.3)	34 (8.0)	0.079	32 (7.5)	34 (8.0)	0.018
Dementia	26861 (13.3)	62 (14.6)	0.037	68 (16.0)	62 (14.6)	0.039
Chronic pulmonary disease	8368 (4.2)	35 (8.3)	0.171	44 (10.4)	35 (8.3)	0.073
Rheumatic disease	4194 (2.1)	10 (2.4)	0.019	7 (1.7)	10 (2.4)	0.050
Peptic ulcer	7915 (3.9)	31 (7.3)	0.147	31 (7.3)	31 (7.3)	<0.001
Liver disease	9324 (4.6)	24 (5.7)	0.047	31 (7.3)	24 (5.7)	0.067
DM without complications	30028 (14.9)	50 (11.8)	0.092	57 (13.4)	50 (11.8)	0.050
DM with complications	5628 (2.8)	10 (2.4)	0.027	11 (2.6)	10 (2.4)	0.015
Hemiparaplegia	967 (0.5)	0 (0.0)	0.098	2 (0.5)	0 (0.0)	0.097
Renal disease	8508 (4.2)	18 (4.2)	0.001	21 (5.0)	18 (4.2)	0.034
Malignancy	8426 (4.2)	37 (8.7)	0.186	35 (8.3)	37 (8.7)	0.017
Metastatic cancer	953 (0.5)	10 (2.4)	0.160	8 (1.9)	10 (2.4)	0.033
Antibiotic use within 7 days of surgery	186699 (92.7)	424 (100)	0.398	386 (91.0)	424 (100)	0.444
Mechanical ventilation within 7 days of surgery	784 (0.4)	0 (0.0)	0.088	1 (0.2)	0 (0.0)	0.069
Catecholamine use within 7 days of surgery	11526 (5.7)	30 (7.1)	0.055	26 (6.1)	30 (7.1)	0.038
Transfusion within 7 days of surgery	54494 (27.0)	131 (30.9)	0.085	131 (30.9)	131 (30.9)	<0.001
Renal replacement therapy within 7 days of surgery	661 (0.3)	1 (0.2)	0.017	2 (0.5)	1 (0.2)	0.040
High intensity care unit admission within 7 days of surgery	8470 (4.2)	14 (3.3)	0.047	10 (2.4)	14 (3.3)	0.057
Fracture location			0.059			0.078
Neck	104352 (51.8)	232 (54.7)		222 (52.4)	232 (54.7)	
Intertrochanteric	92156 (45.7)	182 (42.9)		187 (44.1)	182 (42.9)	
Subtrochanteric	4968 (2.5)	10 (2.4)		15 (3.5)	10 (2.4)	
Type of surgery			0.032			0.049
Open reduction and internal fixation	131393 (65.2)	270 (63.7)		280 (66.0)	270 (63.7)	
Hemiarthroplasty	70083 (34.8)	154 (36.3)		144 (34.0)	154 (36.3)	
Anesthesia time category, min			0.113			0.078
-120	140345 (69.7)	310 (73.1)		322 (75.9)	310 (73.1)	
121–180	46834 (23.2)	94 (22.2)		81 (19.1)	94 (22.2)	
181–240	9857 (4.9)	15 (3.5)		15 (3.5)	15 (3.5)	
241-	4440 (2.2)	5 (1.2)		6 (1.4)	5 (1.2)	
Transfusion volume category, mL			0.124			0.090
0	126060 (62.6)	243 (57.3)		240 (56.6)	243 (57.3)	
1–200	6090 (3.0)	12 (2.8)		12 (2.8)	12 (2.8)	
201–400	32747 (16.3)	77 (18.2)		88 (20.8)	77 (18.2)	
401–600	23752 (11.8)	55 (13.0)		55 (13.0)	55 (13.0)	
601-	12827 (6.4)	37 (8.7)		29 (6.8)	37 (8.7)	
Interval between admission and surgery, days, mean (SD)	5.3 (7.3)	5.7 (5.3)	0.066	5.7 (4.7)	5.7 (5.3)	0.009
Hospital volume of surgery for hip fractures/year, mean (SD)	224 (135)	215 (156)	0.060	220.8 (124.1)	215.2 (156.1)	0.040
Fiscal year			0.147			0.022
2010	29851 (14.8)	70 (16.5)		73 (17.2)	70 (16.5)	
2011	51846 (25.7)	121 (28.5)		120 (28.3)	121 (28.5)	
2012	61030 (30.3)	101 (23.8)		102 (24.1)	101 (23.8)	
2013	58749 (29.2)	132 (31.1)		129 (30.4)	132 (31.1)	
Academic hospital	9766 (4.8)	12 (2.8)	0.105	10 (2.4)	12 (2.8)	0.030
Transferred by ambulance	104264 (51.8)	217 (51.2)	0.011	213 (50.2)	217 (51.2)	0.019
Number of beds categories			0.109			0.079
-200	27331 (13.6)	61 (14.4)		61 (14.4)	61 (14.4)	
201–400	86723 (43.0)	164 (38.7)		167 (39.4)	164 (38.7)	
401–600	61855 (30.7)	149 (35.1)		153 (36.1)	149 (35.1)	
601–800	18835 (9.3)	37 (8.7)		35 (8.3)	37 (8.7)	
801-	6732 (3.3)	13 (3.1)		8 (1.9)	13 (3.1)	

Numbers and percentages are presented unless otherwise stated. SD, standard deviation; SMD, standardized mean difference; BMI, body mass index; DM, diabetes mellitus.

**Table 2:** Outcomes before and after propensity score matching.

	Prematching cohort			Propensity score matched cohort		
	Control	Kampo	*P*	Control	Kampo	*P*
	*n* = 201476	*n* = 424		*n* = 424	*n* = 424	
In-hospital death, *n* (%)	3552 (1.8)	8 (1.9)	0.852	6 (1.4)	8 (1.9)	0.394
Infectious complications, *n* (%)	4647 (2.3)	12 (2.8)	0.578	6 (1.4)	12 (2.8)	0.234
Hospital-acquired pneumonia, *n* (%)	2830 (1.4)	6 (1.4)		5 (1.2)	6 (1.4)	
Surgical site infection, *n* (%)	1239 (0.6)	3 (0.7)		1 (0.2)	3 (0.7)	
Sepsis, *n* (%)	710 (0.4)	4 (0.9)		2 (0.5)	4 (0.9)	

## Data Availability

Data cannot be made publicly available for ethical reasons as the data are patient data. The data are available to interested researchers upon request to the corresponding author, pending ethical approval.
